# Dietary Transfer of Zinc Oxide Nanoparticles Induces Locomotive Defects Associated with GABAergic Motor Neuron Damage in *Caenorhabditis elegans*

**DOI:** 10.3390/nano13020289

**Published:** 2023-01-10

**Authors:** Chun Ming How, Chi-Wei Huang

**Affiliations:** 1 Department of Bioenvironmental Systems Engineering, National Taiwan University, Taipei 10617, Taiwan; 2 Department of Marine Environmental Engineering, National Kaohsiung University of Science and Technology, Kaohsiung 81157, Taiwan

**Keywords:** ZnO nanoparticles, trophic transfer, neurotoxicity, GABAergic motor neuron, neuron damage

## Abstract

The widespread use of zinc oxide nanoparticles (ZnO-NPs) and their release into the environment have raised concerns about the potential toxicity caused by dietary transfer. However, the toxic effects and the mechanisms of dietary transfer of ZnO-NPs have rarely been investigated. We employed the bacteria-feeding nematode *Caenorhabditis elegans* as the model organism to investigate the neurotoxicity induced by exposure to ZnO-NPs via trophic transfer. Our results showed that ZnO-NPs accumulated in the intestine of *C. elegans* and also in *Escherichia coli* OP50 that they ingested. Additionally, impairment of locomotive behaviors, including decreased body bending and head thrashing frequencies, were observed in *C. elegans* that were fed *E. coli* pre-treated with ZnO-NPs, which might have occurred because of damage to the D-type GABAergic motor neurons. However, these toxic effects were not apparent in *C. elegans* that were fed *E. coli* pre-treated with zinc chloride (ZnCl_2_). Therefore, ZnO-NPs particulates, rather than released Zn ions, damage the D-type GABAergic motor neurons and adversely affect the locomotive behaviors of *C. elegans* via dietary transfer.

## 1. Introduction

Engineered nanoparticles (ENPs), which are extensively used in industrial and domestic products, are emitted into the environment [1]. This has raised concerns due to their potential toxicity to biota [1,2]. Zinc oxide nanoparticles (ZnO-NPs), with a global production of approximately 550–33,400 tons, are one of the most widely used ENPs in the electrical and manufacturing industries and personal care products [3,4]. In addition, due to their biocompatibility and cost-effectiveness, ZnO-NPs have been increasingly used in the biomedical field, such as in antibacterial, antifungal, antiviral, antidiabetic, and wound healing applications [5]. These advantages have also resulted in the growing usage of ZnO-NPs in dentistry [6]. Moreover, ZnO-NPs exhibit luminescent properties, making ZnO-NPs an excellent candidate for bioimaging [7]. ZnO-NPs are also commonly used as medicine in livestock, aquaculture, and pet animals despite the occasionally occurring side effects [8]. ZnO-NPs are also used in the synthesis of hybrid nanomaterials, which show excellent sorbent properties for the removal of pollutants [9,10,11]. As a result, the production, manufacturing, and consumption of ZnO-NPs result in their emission via industrial effluents [12]. The amount of ZnO-NPs entering sewage treatment plants by the European Union is estimated to be 1.05 million kg per year (1.7–45 μg/L in the effluent of sewage treatment plants) [12,13]. Considering that environmental ZnO-NPs levels are likely to rise due to the growing demand [13], this contaminant is a cause for concern because of its potential adverse effects on biota [14].

Dietary transfer is an important pathway for pollutant transfer and accumulation, which might cause harmful effects in organisms at higher trophic levels [15]. The inclusion of dietary transfer as a factor in ecotoxicological risk assessment for aquatic species is recommended considering the significant toxicity caused by dietary exposure [16]. Due to the long-lasting and non-biodegradable properties of metals, the effects of the accumulation of metal-based ENPs due to dietary transfer require urgent investigation [2]. ENPs, such as ZnO, silver (Ag), and titanium dioxide (TiO_2_), are transferred through food chains and accumulate in the higher trophic levels [17,18,19]. The enhanced accumulation of metal-based ENPs, including Ag, silicon dioxide (SiO_2_), tin oxide (SnO_2_), cerium oxide (CeO_2_), and magnetite (Fe_3_O_4_), has several sublethal adverse effects on reproduction, development, and locomotion [20,21,22]. Although ZnO-NPs are one of the most widely used ENPs, studies related to their toxic effects induced by dietary intake are limited. In addition, whether ZnO-NPs from dietary transfer affect other target tissues, such as the neurons, remains unclear.

Metal-based ENPs accumulate in various organs or tissues after ingestion and induce toxicity predominantly via reactive oxygen species (ROS) [23]. Recent research has suggested that neuromuscular defects are induced by ENPs in the nematode *Caenorhabditis elegans* [24,25]. Furthermore, locomotion in zebrafish and *C. elegans* is known to be adversely affected by ZnO-NPs [26,27]. Previous studies showed that D-type GABAergic motor neurons, which control locomotive behaviors, are potential targets of toxicants, including quantum dots and heavy metals [28,29]. The altered behaviors and neurotoxicity may reduce the fitness of organisms and result in ecotoxicity [30]. However, the involvement of the dietary transfer of ZnO-NPs in neurotoxicity and its underlying mechanisms remain unknown.

To investigate the toxic effects and mechanisms of the dietary transfer of ZnO-NPs, we used *C. elegans* as the model organism and *Escherichia coli* OP50 as its food source to establish a dietary transfer assay model. *C. elegans* is regarded as a useful model in environmental toxicology and neurotoxicology due to its short life cycle, ease of maintenance, and availability of mutants and transgenic strains [31]. A 72 h exposure period, which covers all developmental stages until puberty, may be considered long-term exposure in *C. elegans* [32]. Our study aims to investigate the toxic effects of the long-term dietary transfer of ZnO-NPs in the *E. coli*–*C. elegans* food chain model by examining their accumulation in these organisms and assessing the impairment of locomotive behaviors and D-type GABAergic motor neurons.

## 2. Materials and Methods

### 2.1. Chemicals, Characterization, and Strains Maintenance

All of the chemicals used in this study were purchased from Sigma-Aldrich Chemicals Co. (St. Louis, MO, USA). Zinc chloride (ZnCl_2_) was used as a control to distinguish the effects of dissolved zinc ions (Zn^2+^). ZnO-NPs (<50 nm, assay grade, >97% purity) were sonicated and freshly prepared in 1000 mg/L stock solution before the assays. Characteristics of ZnO-NPs, including morphology and hydrodynamic diameter, have been previously documented [26]. The TEM image of ZnO-NPs used in the present study was shown in Figure S1. A previous study also reported the TEM image and X-ray diffraction (XRD) results of ZnO-NPs [33]. The concentrations of Zn released from ZnO-NPs in a Luria–Bertani broth (LB) medium during the 8 h *E. coli* exposure period were determined at 0, 4, 6, and 8 h at 37 °C. The initial concentration of the ZnO-NPs suspension prepared in the LB medium was 5 mg/L. The samples were filtered through Amicon Ultra-15 Ultracel-3 centrifuge tubes (3 kDa cutoff ≈ 0.9 nm, Millipore, Billerica, MA, USA) to remove the remaining ZnO-NPs, and the concentrations of Zn^2+^ in the aqueous phase were measured using inductively coupled plasma atomic emission spectroscopy (ICP-AES, Spectro Ciros 120, Kleve, Germany).

*E. coli* OP50 and *C. elegans* strains, including wild-type N2 and transgenic EG1285 strain [*unc-47*p::GFP + *lin-15*(+)], were purchased from the *Caenorhabditis* Genetics Center (CGC, University of Minnesota, MN, USA). The freshly prepared overnight bacterial culture from a single colony was used for ZnO-NP exposure and dietary transfer assay. For maintaining the *C. elegans* strains, all of the worms were cultured at 20 °C on a nematode growth medium (NGM) agar plate covered with a bacterial lawn of *E. coli* OP50.

### 2.2. Measurement of Zn Concentration in E. coli OP50

Saturated *E. coli* OP50 were freshly prepared and subcultured in the LB medium with various concentrations of ZnO-NPs for 8 h at 37 °C according to the methods of a previous study [20]. Subsequently, the bacterial culture was washed three times using deionized water by centrifugation (15 min at 3000× *g*). The *E. coli* OP50 pellet that was collected was digested using concentrated nitric acid (HNO_3_), and the Zn concentration was analyzed using ICP-AES.

### 2.3. Dietary Transfer Assay

The dietary transfer assay was adapted from a previous study with minor modifications [20]. *E. coli* OP50 treated with ZnO-NPs or ZnCl_2_ for 8 h were washed and re-suspended in deionized water. Subsequently, they were spread on the NGM plates onto which the *C. elegans* L1-larvae were placed. After 72 h of exposure, the worms were washed using M9 buffer for future assays.

To characterize the distribution and localization of ZnO-NPs in *C. elegans*, rhodamine B (RhoB) was used to label the ZnO-NPs, and unbound RhoB was removed by performing dialysis for 24 h in deionized water (regenerated cellulose dialysis tubing, MWCO: 6000–8000; Orange Scientific, Braine-l’Alleud, Belgium) [34]. The freshly prepared RhoB-labeled ZnO-NPs (RhoB/ZnO-NPs) were administered to the *E. coli* OP50 for 8 h. The RhoB and deionized water without ZnO-NPs (RhoB/deionized water) were treated using dialysis for 24 h and used as the control. Wild-type N2 *C. elegans* L1-larvae were fed with RhoB/ZnO-NPs or RhoB pre-treated *E. coli* OP50 for 96 h. After following the exposure and washing steps, the worms were anesthetized in 50 mM sodium azide on 2% agarose gel mounted on a glass slide. Fluorescent images of the worms were acquired using an epifluorescence microscope (Leica, Wetzlar, Germany) with a suitable filter set (excitation, 550 ± 30 nm; emission, 615 ± 45 nm) and a digital camera. Fluorescence intensities were analyzed and quantified using ImageJ [35]. The tests were conducted at least three times independently, and 25 worms were scored per treatment in each replicate.

### 2.4. Locomotive Behaviors Tests

Wild-type N2 *C. elegans* were washed after exposure via dietary transfer and subjected to locomotive behavior tests. Locomotive behaviors, including body bending and head thrashing frequencies, were studied by modifying the methods used in a previous study [36]. The frequencies of body bending during 20 s and head thrashing during 1 min were manually measured and calculated. The tests were conducted at least three times independently, and 20 worms were scored per treatment in each replicate.

### 2.5. GABAergic Neuron Toxicity Assay

Transgenic EG1285 strain *C. elegans* were washed after exposure through dietary transfer. The morphology of green fluorescent protein (GFP)-labeled GABAergic neurons was characterized using an epifluorescence microscope. Fluorescent images of the worms were captured for the determination of neuron cell and gap numbers in GABAergic D-type motor neurons. The abnormality of GABAergic motor neurons (%) was defined as the total counts of GABAergic motor neuron cell loss and gap numbers divided by the total GABAergic motor neuron cells and the connections between the neuron cells in normal conditions. The tests were conducted at least three times independently, and 20 worms were scored per treatment in each replicate.

### 2.6. Statistical Analysis

SPSS Statistics for Windows, version 22.0 (IBM Corp., Armonk, NY, USA) was used to conduct the statistical analysis. The data were expressed as the mean ± standard error of the means (SEM). Statistical significance was determined using a one-way analysis of variance (ANOVA) with a least significant difference (LSD) post-hoc test and indicated using different lowercase letters (*p* < 0.05).

## 3. Results and Discussion

### 3.1. Concentration of Zn Released from ZnO-NPs in LB Medium

**Figure 1:**
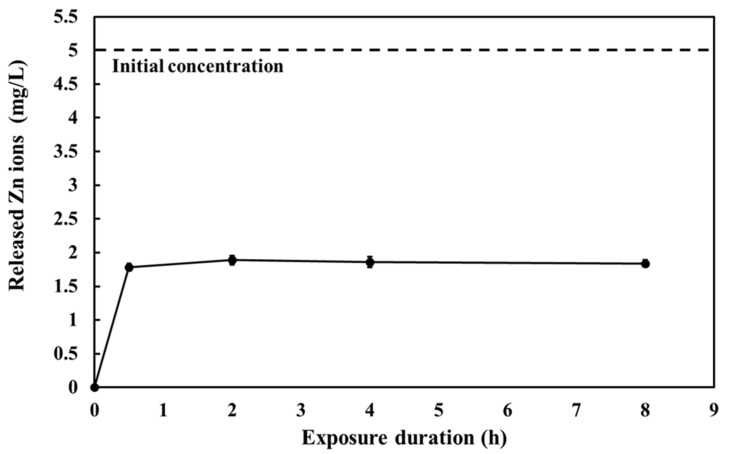
Released Zn ions from ZnO-NPs in LB medium. Initial concentration of ZnO-NPs suspension was 5 mg/L prepared in LB medium. Samples were incubated at 37 °C and analyzed at different time points at 0, 0.5, 2, 4, and 8 h. Samples were filtered to remove undissolved ZnO-NPs, and then the concentrations of Zn ions in the aqueous phase were measured using ICP-AES. The data are shown as the mean ± SEM.

Metal nanoparticles exhibit distinct characteristics under different environmental conditions because of their interactions with abiotic and biotic factors [37]. Additionally, the ZnO-NPs are substantially affected by the background medium, resulting in the release of varying amounts of Zn^2+^ [26]. Therefore, before *E. coli* exposure, we determined the concentrations of Zn^2+^ released from the ZnO-NPs in the LB medium at different time points. The Zn^2+^ concentration in the LB medium remained constant at approximately 1.8 mg/L after 0.5, 2, 4, and 8 h (Figure 1). Furthermore, the Zn^2+^ concentration reached a steady state immediately after the ZnO-NPs were added to the LB medium (Figure 1).

Figure 1 shows that Zn^2+^ constituted approximately 35% of the total ZnO-NPs in the LB medium, implying that compared to the concentration of the particulate form, that of the ionic form of ZnO-NPs was relatively lower in the LB medium. Recent studies have also demonstrated the low solubility and stability of ZnO-NPs in the LB medium (5–51% Zn^2+^ of total ZnO-NPs) [38,39], which may have been due to the presence of organic matter in the LB medium. Some types of organic matter may inhibit the release of free Zn^2+^ from ZnO-NPs [40,41]. Moreover, the dissolution rate of ZnO-NPs is inversely correlated to aliphatic carbon content and hydrogen/carbon ratio [42]. Therefore, both ionic and particulate forms of ZnO-NPs can potentially transfer to *E. coli* OP50, and the accumulation was further investigated.

### 3.2. Zn Accumulation in E. coli OP50

To investigate the accumulation of ZnO-NPs in *E. coli* OP50 that might further transfer to *C. elegans*, we exposed *E. coli* OP50 to different concentrations of ZnO-NPs (5, 10, 50, and 100 mg/L) and analyzed the Zn concentrations in the bacteria. The concentrations were designed based on the minimal inhibitory concentration (MIC) of 400 mg/L for ZnO-NPs in *E. coli* strain [43]. We selected concentrations below the MIC, and serial dilution was applied to establish the dose-response relationships for the toxicological endpoints.

**Figure 2:**
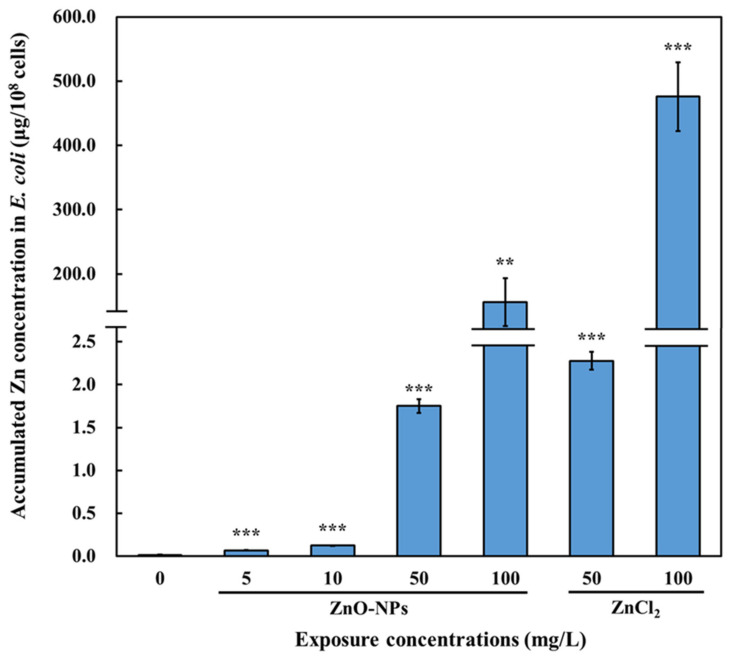
Accumulated Zn concentration in *E. coli* OP50 upon ZnO-NP or ZnCl_2_ exposure. Saturated *E. coli* OP50 were diluted and incubated in LB medium with various concentrations of ZnO-NPs or ZnCl_2_ for 8 h at 37 °C. Subsequently, *E. coli* OP50 pellet was washed and collected for Zn concentration analysis using ICP-AES. The data are shown as the mean ± SEM. Statistical significance was determined by ANOVA with LSD post-hoc test to compare to the control (0 mg/L). (**: *p* < 0.01, ***: *p* < 0.001).

We found that Zn concentrations in bacterial cells increased in a dose-dependent manner (Figure 2). Exposure to 100 mg/L of ZnO-NPs caused a cellular burden of approximately 200 µg/10^8^ cells, which was 100 times higher than that due to 50 mg/L of ZnO-NP exposure (Figure 2). Exposure to ZnCl_2_ also resulted in a substantial accumulation of Zn in the bacterial cells; 50 mg/L ZnCl_2_ led to a cellular burden of approximately 2.5 µg/10^8^ cells, whereas 100 mg/L ZnCl_2_ caused a cellular burden of approximately 500 µg/10^8^ cells (Figure 2).

Thus, ZnO-NPs accumulated in *E. coli* OP50 after 8 h of exposure at all of the examined concentrations (Figure 2). Metal nanoparticles, such as TiO_2_ and Ag, accumulated in the bacteria, including *E. coli* and *Pseudomonas aeruginosa*, and then were transferred to higher trophic levels [20,44]. Several studies have shown that ZnO-NPs can damage the bacterial cell wall and enhance membrane permeability, thereby resulting in their accumulation in bacteria [45,46]. Additionally, the internalization of ZnO-NPs by *E. coli* and other bacterial cells has been observed previously [47,48]. The high concentration (100 mg/L) of ZnO-NPs and ZnCl_2_ largely increased the Zn accumulation compared with 50 mg/L, which may be due to the membrane damage that facilitated higher Zn accumulation in the cytoplasm [49]. Therefore, our results suggest that ZnO-NPs can accumulate in prey (*E. coli*) and potentially be transferred to higher trophic levels through dietary intake.

### 3.3. Distribution and Accumulation of ZnO-NPs in C. elegans via Dietary Transfer

**Figure 3:**
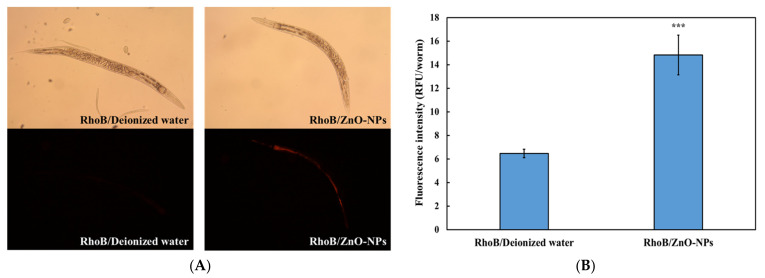
Accumulated ZnO-NPs in *C. elegans* through dietary transfer. Wild-type N2 *C. elegans* L1-larvae were fed with rhodamine B (RhOB)-labeling ZnO-NPs (RhoB/ZnO-NPs) pretreated *E. coli* OP50 for 96 h. RhoB/Deionized water was used as the control. After exposure and washing, (**A**) fluorescence images of worms were taken, and (**B**) fluorescence intensity was analyzed using ImageJ. The data are shown as the mean ± SEM. The tests were conducted at least three times independently, and 25 worms were scored per treatment in each replicate. Statistical significance was determined by ANOVA with LSD post-hoc test to compare to the control. (***: *p* < 0.001).

To further assess the dietary transfer of ZnO-NPs from *E. coli* to *C. elegans*, the worms were exposed to *E. coli* OP50 pre-treated with ZnO-NPs. The control worms were fed with *E. coli* pre-treated with RhoB/deionized water. The fluorescent dye RhoB was used to label the ZnO-NPs to visualize the distribution and accumulation of the ZnO-NPs due to dietary transfer. Compared with the controls (RhoB/deionized water), the RhoB-labeled ZnO-NPs accumulated mainly in the pharynx and intestine of *C. elegans* (Figure 3A). Quantification of the fluorescence intensity showed that the background levels in the controls were approximately 6 RFU/worm, which significantly increased to approximately 15 RFU/worm in the presence of RhoB-labeled ZnO-NPs (Figure 3B).

While the dietary transfer of ZnO-NPs in aquatic food chains has been demonstrated in several studies [19,50,51,52], little is known about their distribution. Trophic transfer of ZnO-NPs occurs in simple food chains involving algae (*Chlorella ellipsoidea*) and clams (*Corbicula fluminea*) [53]. Furthermore, goldfish fed with brine shrimp pre-exposed to ZnO-NPs showed significant accumulation of Zn in the intestine [54]. In *C. elegans*, the intestine is the primary target of nanomaterials, including SiO_2_, carbon nanotubes, graphene oxide, and Ag [20,55,56,57]. This may be the reason for the significant ZnO-NP accumulation in the intestine and pharynx of *C. elegans* due to dietary transfer from *E. coli* OP50 (Figure 3A).

### 3.4. Effects of Dietary Transfer of ZnO-NPs on Locomotive Behaviors of C. elegans

The sublethal endpoints, including body bending and head thrashing frequencies, of *C. elegans* have been used to assess neurotoxicity [31,58,59]. Moreover, we previously found that aquatic exposure to ZnO-NPs in simulated surface water (EPA water) significantly impairs locomotive behaviors, indicating that neurotoxicity is a potential result of ZnO-NP exposure [26]. Therefore, the effects of the dietary transfer of ZnO-NPs on *C. elegans* were investigated using locomotive behavior tests. In addition, ZnCl_2_ was used to differentiate between toxicity due to ionic Zn and that caused by ZnO-NPs.

**Figure 4:**
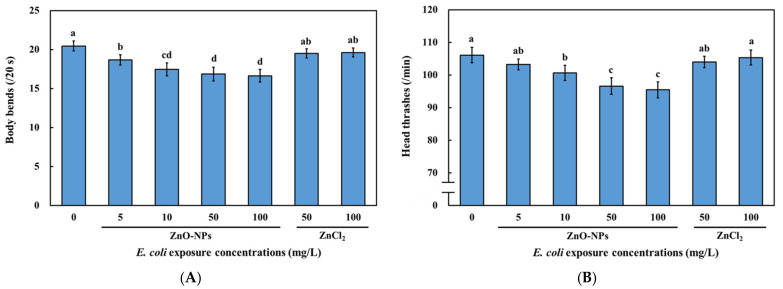
Locomotive behavior defects resulted from dietary transfer of ZnO-NPs in *C. elegans.* Wild-type N2 *C. elegans* L1-larvae were fed with ZnO-NPs or ZnCl_2_ pretreated *E. coli* OP50 for 72 h. After exposure and washing, (**A**) body bends and (**B**) head thrashes of worms were determined. The data are shown as the mean ± SEM. The tests were conducted at least three times independently, and 20 worms were scored per treatment in each replicate. Statistical significance was determined by one-way ANOVA with LSD post-hoc test and indicated by different lowercase letters (*p* < 0.05).

The body bending frequency decreased in *C. elegans* fed with ZnO-NPs pre-treated *E. coli* OP50 in a dose-dependent manner (Figure 4A). In contrast, there was no significant difference in the body bending frequency of *C. elegans* fed with relatively high concentrations (50 and 100 mg/L) of ZnCl_2_ pre-treated *E. coli* OP50 and that of the controls (fed with 0 mg/L ZnCl_2_ pre-treated *E. coli* OP50) (Figure 4A). Tests pertaining to the head thrashing frequency exhibited similar results (Figure 4B). *E. coli* pre-treated with 50 and 100 mg/L of ZnO-NPs demonstrated a 9–10% reduction in head thrashing and a 17–18% reduction in body bending (Figure 4A,B), implying that body bending frequency may be a more sensitive endpoint than head thrashing.

Our results suggest that the predicted environmental concentration of ZnO-NPs (76 μg/L) [60] may be harmful to locomotive behavior and cause ecotoxicity. Altered behaviors caused by environmental toxicants can reduce the fitness and population of organisms, indicating the potential impact of neurotoxicity on the ecosystem [30]. A previous study showed that exposure to ZnO-NPs impaired motor functions in mice [11]. Liquid exposure to ZnO-NPs shows impairment of locomotive behaviors in *C. elegans* and zebrafish, which is more significant than that caused by ZnCl_2_ [26,27]. Thus, the biological actions of ZnO-NPs and Zn^2+^ are different, and the impairment of motor functions induced by ZnO-NPs may be similar in different species. It has been shown that the sensitivity of body bends to the liquid exposure of ZnO-NPs was higher than head thrashes in *C. elegans*, which is in agreement with our results [26]. Similarly, the direct liquid exposure of 500 μg/L of ZnO-NPs caused higher toxic effects on body bends than 500 μg/L of ZnCl_2_ [26]. Moreover, our results showed that impaired locomotive behaviors and neurotoxicity in *C. elegans* were due to the dietary transfer of ZnO-NPs (Figure 4A,B). The dietary transfer of Ag nanoparticles is known to disrupt the locomotion of springtails (Collembola) [21]. Additionally, copper (Cu) nanoparticles accumulate in *Daphnia magna* by dietary transfer and impair their feeding rate [61]. Therefore, various metal nanoparticles in the environment can produce toxic effects when they are transferred to higher trophic levels. Although the dietary transfer of ZnO-NPs through trophic levels has been reported, its neurotoxicity due to dietary transfer has rarely been reported. Our findings suggest that ZnO-NPs can accumulate in *C. elegans* via trophic transfer, thereby impairing locomotion behaviors. However, the mechanisms behind the locomotion defects due to the dietary transfer of metal-based nanoparticles are unclear.

### 3.5. Effects of Dietary Transfer of ZnO-NPs on D-Type GABAergic Motor Neurons of C. elegans

**Figure 5:**
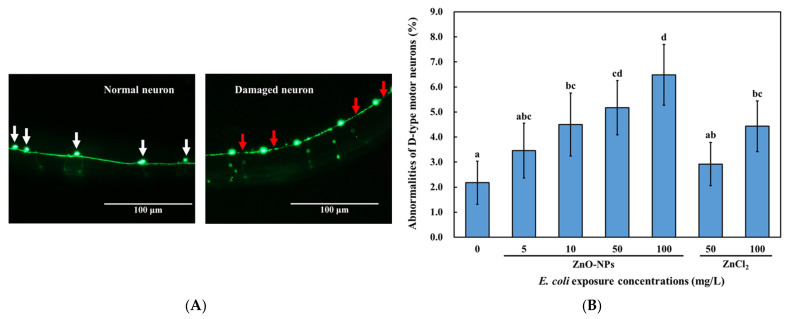
GABAergic neuron damage resulted from dietary transfer of ZnO-NPs in *C. elegans.* Transgenic strain EG1285 (*unc-47*p::GFP) L1-larvae were fed with ZnO-NPs or ZnCl_2_ pretreated *E. coli* OP50 for 72 h. After exposure and washing, fluorescence images of worms were taken to determine neural abnormalities, including neuron loss and degenerating commissures of GABAergic D-type motor neurons. (**A**) Representative image for determination of GABAergic neuronal damage. White arrows indicate normal cell bodies; red arrows indicate gaps on the neuronal cord. (**B**) GABAergic neuron damage resulted from dietary transfer of ZnO-NPs. The data are shown as the mean ± SEM. The tests were conducted at least three times independently, and 20 worms were scored per treatment in each replicate. Statistical significance was determined by one-way ANOVA with LSD post-hoc test and indicated by different lowercase letters (*p* < 0.05).

The D-type GABAergic motor neurons are inhibitory motor neurons that control locomotive behaviors in *C. elegans* [31,62]. Therefore, we investigated the effect of the dietary transfer of ZnO-NPs on these neurons (Figure 5A) and found that they decreased the cell bodies of D-type GABAergic motor neurons in a dose-dependent manner (Figure 5B). The representative images of GABAergic neuron in *C. elegans* with different treatments were shown in Figure S2. In addition, ZnO-NP treatment caused greater degeneration of D-type GABAergic motor neurons than that caused by ZnCl_2_ (Figure 5B). Moreover, a gap formation on the cords of the D-type GABAergic motor neurons increased due to the dietary exposure of ZnO-NPs in a dose-dependent manner, which was more significant than that caused by the ZnCl_2_ treatment (Figure 5B). Thus, dietary exposure to ZnO-NPs significantly damages D-type GABAergic neurons in *C. elegans*. In addition, we have checked the cholinergic motor neurons, which are the other motor neurons controlling the locomotive behavior of *C. elegans*. The results of the gap numbers showed that there is no significant difference between the control and treatment with ZnO-NPs or ZnCl_2_ (Figure S3).

Toxic metals and nanoparticles, such as quantum dots, have been shown to adversely affect locomotive behaviors and damage D-type GABAergic motor neurons [28,29]. Nevertheless, other nanomaterials, including graphene-based Ag ones, do not harm GABAergic neurons [25,55]. This may be because of different toxicity mechanisms. Our results confirmed that the D-type GABAergic motor neuron system was damaged in *C. elegans* that were fed with ZnO-NPs pre-treated *E. coli* OP50 (Figure 5B), which might have contributed to the impairment of the locomotive behaviors (Figure 4A,B). A previous study found that exposure to ZnO-NPs also induced dopaminergic neuronal damage in zebrafish brains [63]. Additionally, exposure to ZnO-NPs resulted in neuronal damage in the brains of Wistar rats [64]. These suggest that ZnO-NP-induced neuronal damage might not be exclusive to *C. elegans*. A previous study showed that cancer cell lines were more sensitive to the cytotoxic effects of ZnO-NPs than normal cell lines [65]. Despite the growing body of evidence demonstrating the promising potential of ZnO-NPs in biomedical applications, the non-selective cytotoxic effects against the various cell lines of ZnO-NPs remain controversial [66]. Our findings suggest that precautions should be taken as dietary ZnO-NPs might result in neuronal damage in vivo.

Notably, the adverse effects on the neuron system and locomotive behaviors were not observed in the *C. elegans* that were fed ZnCl_2_ pre-treated *E. coli* OP50 (Figure 4 and Figure 5), indicating that the effects of the dietary transfer of ZnO-NPs were primarily due to particulate forms rather than Zn^2+^. The particle-specific effects of the dietary transfer of ZnO-NPs were also observed in several studies, especially those on bioaccumulation [19,51]. The present study further suggests that neurotoxicity caused by the dietary transfer of ZnO-NPs is more significant than that due to ZnCl_2_ exposure. Previous studies have mainly reported the accumulation of dietary ZnO-NPs in the gut, as well as mortality, reproduction, and oxidative stress response [19,50,51,52]. We provided evidence that dietary ZnO-NPs can also adversely affect the locomotion of *C. elegans* by causing neuronal damage in vivo. Therefore, our findings reveal rarely reported neuronal damage induced by dietary ZnO-NP exposure.

## 4. Conclusions

In summary, the present study provides evidence regarding the dietary transfer of ZnO-NPs from *E. coli* OP50 to *C. elegans* and their toxic effects on locomotive behaviors and D-type GABAergic motor neurons. Additionally, these effects were found to be more significant in *C. elegans* that were fed ZnO-NPs pre-treated *E. coli* OP50 than in those exposed to ZnCl_2_ pre-treated *E. coli* OP50, which indicates that the neurotoxicity caused by the dietary transfer of ZnO-NPs is mostly due to nanoparticles rather than Zn^2+^. The results of the present study highlight the potential neurotoxicity and toxic mechanisms of ZnO-NPs transferred through food chains.

## Data Availability

The data would be available on request to the corresponding author.

## Supplementary Materials

The following supporting information can be downloaded at: https://www.mdpi.com/article/10.3390/nano13020289/s1, Figure S1: TEM image of ZnO nanoparticles (ZnO-NPs) used in this study; Figure S2 Representative images of GABAergic neurons in *C. elegans* (A–C); Figure S3. Effects of ZnO-NPs and ZnCl2 on cholinergic motor neurons.
