# Supplementary material for: Dietary Transfer of Zinc Oxide Nanoparticles Induces Locomotive Defects Associated with GABAergic Motor Neuron Damage in Caenorhabditis elegans
Source: Nanomaterials (Basel). 2023 Jan 10;13(2):289. doi: 10.3390/nano13020289 (PMC9866546; doi:10.3390/nano13020289)

Figure S1. TEM image of ZnO nanoparticles (ZnO-NPs) used in this study

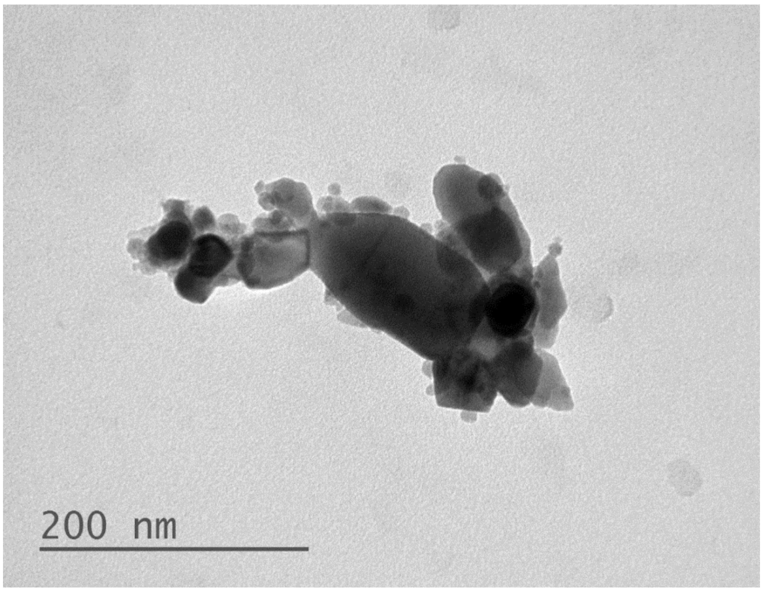

Figure S2 Representative images of GABAergic neurons in *C. elegans*（A－C）

(A) Control

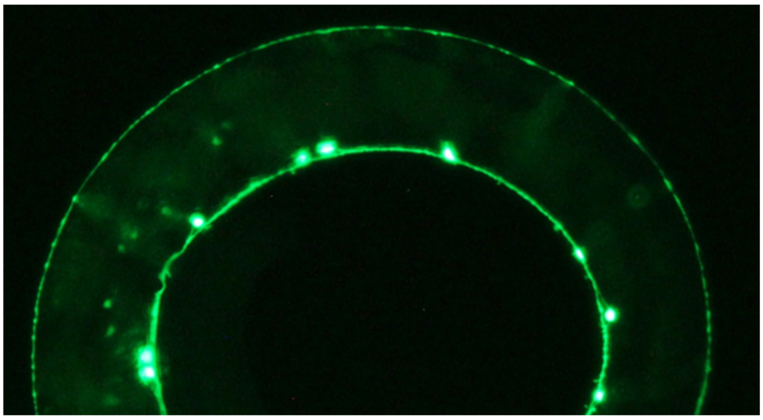

(B) Fed on *E. coli* pretreated with 50 mg/L $ZnCl_2$

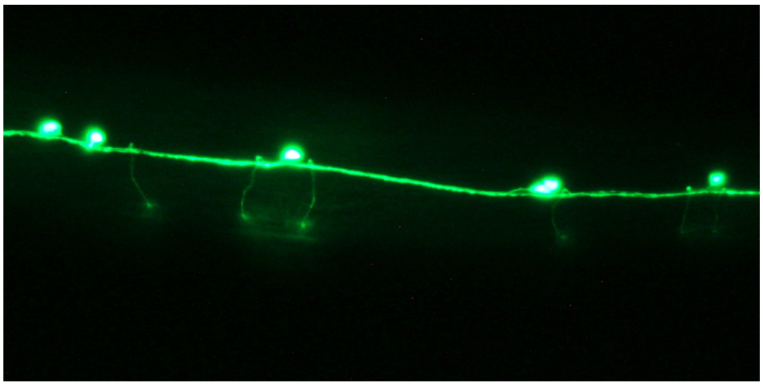

(C) Fed on *E. coli* pretreated with 50 mg/L ZnO-NPs

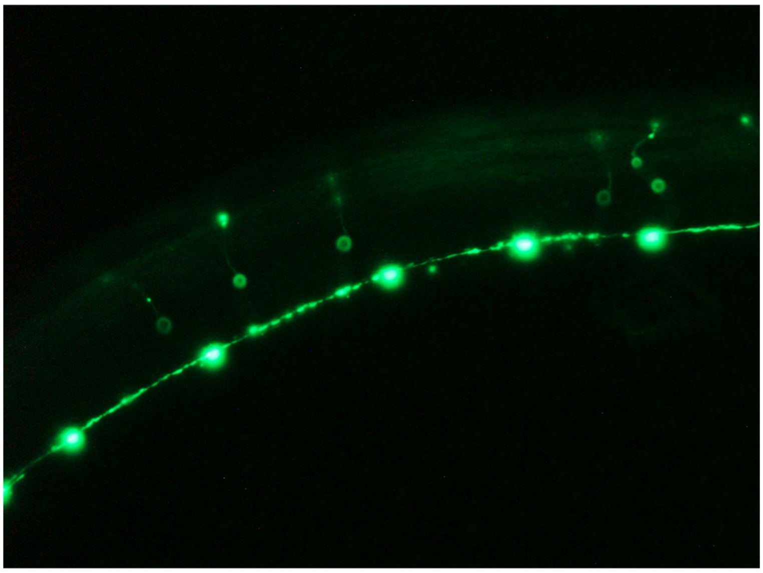

Figure S3. Effects of ZnO-NPs and $ZnCl_2$ on cholinergic motor neurons

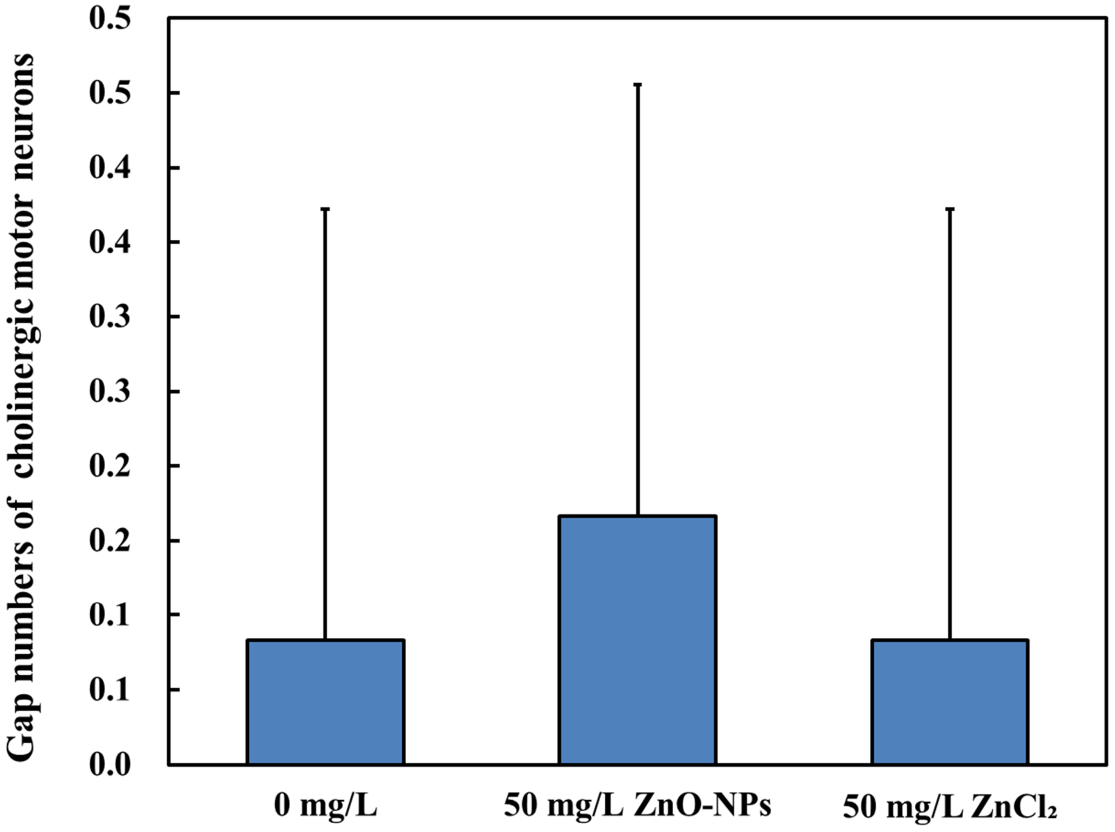

Supplement: Supplementary file 1 [file nanomaterials-13-00289-s001.zip › nanomaterials-2156402-SI.pdf]
